# Mineral-Based Coating of Plasma-Treated Carbon Fibre Rovings for Carbon Concrete Composites with Enhanced Mechanical Performance

**DOI:** 10.3390/ma10040360

**Published:** 2017-03-29

**Authors:** Kai Schneider, Matthias Lieboldt, Marco Liebscher, Maik Fröhlich, Simone Hempel, Marko Butler, Christof Schröfl, Viktor Mechtcherine

**Affiliations:** 1 Technische Universität Dresden, Institute of Construction Materials, DE-01062 Dresden, Germany; kai.schneider@tu-dresden.de (K.S.); Marco.Liebscher@tu-dresden.de (M.L.); Simone.Hempel@tu-dresden.de (S.H.); marko.butler@tu-dresden.de (M.B.); Christof.schroefl@tu-dresden.de (C.S.); 2 Technische Universität Dresden, Institute of Concrete Structures, DE-01062 Dresden, Germany; matthias.lieboldt@tu-dresden.de; 3 Leibniz Institute for Plasma Science and Technology e.V. (INP Greifswald), DE-17489 Greifswald, Germany; maik.froehlich@inp-greifswald.de

**Keywords:** carbon fibre, plasma treatment, concrete, interphase, mineral coating

## Abstract

Surfaces of carbon fibre roving were modified by means of a low temperature plasma treatment to improve their bonding with mineral fines; the latter serving as an inorganic fibre coating for the improved mechanical performance of carbon reinforcement in concrete matrices. Variation of the plasma conditions, such as gas composition and treatment time, was accomplished to establish polar groups on the carbon fibres prior to contact with the suspension of mineral particles in water. Subsequently, the rovings were implemented in a fine concrete matrix and their pull-out performance was assessed. Every plasma treatment resulted in increased pull-out forces in comparison to the reference samples without plasma treatment, indicating a better bonding between the mineral coating material and the carbon fibres. Significant differences were found, depending on gas composition and treatment time. Microscopic investigations showed that the samples with the highest pull-out force exhibited carbon fibre surfaces with the largest areas of hydration products grown on them. Additionally, the coating material ingresses into the multifilament roving in these specimens, leading to better force transfer between individual carbon filaments and between the entire roving and surrounding matrix, thus explaining the superior mechanical performance of the specimens containing appropriately plasma-treated carbon roving.

## 1. Introduction

The development of carbon fibre (CF) reinforced concrete has attracted much attention in recent years due to the emerging possibility of designing and producing sophisticated, thin-walled concrete structures, with extraordinary mechanical performance, very high durability, and enhanced potential for free-form designs in comparison to steel bar reinforced concrete [1,2]. The use of carbon reinforcement in the form of textiles or thin bars is not confined to new construction, but is also specifically interesting with respect to the strengthening and repair of existing structures [3]. However, the mechanical properties of such carbon concrete composites deteriorate dramatically under elevated temperatures, especially in the case of exposure to fire, with the result that key construction markets are still closed to them. A crucial issue is the common use of polymer coatings on the multifilament carbon roving, which constitutes the bonding interphase between (1) individual carbon filaments of the roving; and (2) carbon roving and concrete matrix [4]. Hence, the load-bearing behaviour of the carbon concrete composite is strongly related to the properties of the polymer-based CF coating. These polymer coatings, however, possess very temperature-dependent mechanical properties. Upon a temperature increase, the storage moduli of the widely used thermoplastic or thermosetting polymers decrease to a great extent. This deterioration of the mechanical properties begins as early as when the specific glass transition temperature is reached. At higher temperatures, melting or chemical degradation result in the complete loss of the load-bearing capacity and, hence, of the function of the CF as reinforcement in the concrete composite [5,6,7]. Silva et al. [8] have demonstrated a sudden worsening of load-bearing behaviour above 400 °C, due to deteriorated polymer coatings. 

A promising approach is to replace such polymer-based coatings with reactive, inorganic-based coatings. Badanoiu and Holmgren compared polymer-modified matrices and matrix compositions containing either silica fume (SF) or a combination of polymers and SF. Their results demonstrated improved carbon fibre bonding when only using SF [9]. Similar positive results were attained in the detailed investigation by Nadiv et al. [10], when using a particular type of SF incorporated between the filaments and when reacted in a pozzolanic manner, improving the reinforcing effect when compared to non-infiltrated multifilament yarns. 

However, when using mineral-based coatings, thermodynamic contrarieties need to be mastered. Coating intrinsic non-polar and, consequently, hydrophobic carbon fibres with a suspension of mineral powder in water is a challenging task. While the wetting behaviour of the carbon fibres can be tailored by using an appropriate polymer sizing [11,12,13,14], such a sizing may also lose its functional groups and degrade with increasing temperature [15]. Another possibility to enhance the load-bearing behaviour of carbon fibre reinforced concrete is to develop a denser fibre/matrix interphase, leading to more intense physical bonding and increased fibre pull-out forces due to the larger areas of fibre friction during fibre pull-out from the matrix [16]. 

A very attractive approach for modifying the wetting behaviour of the CF is treating them with plasma to form specific functional groups on the CF surface [17,18,19]. The application of an oxygen plasma results in the appearance of covalently bound, oxygen-based functional groups on the CF surfaces [20]. This approach dramatically improves the wetting behaviour of CFs with highly polar media such as water [21]. 

Until now, plasma techniques have mainly been used to modify CFs for producing composites with polymer matrices [22,23,24,25]. For cement-based matrices, plasma modification of fibres has been tentatively explored, but on polymer fibres and not on carbon yarns [26]. Plasma treatment of polypropylene (PP) fibres to be implemented in concrete resulted in an improved flexural strength and toughness of the cementitious composites [27]. Plasma-treated polyethylene (PE) fibres were also reported to yield increases in fibre pull-out forces, as compared to non-treated references [28,29]. Tosun et al. [30] showed a different flexural performance of PE fibre-reinforced cement composites, depending on the plasma source. The sequence of the present investigation is comprised of several steps and merges the plasma treatment of carbon yarns with the morphological and fracture-mechanical properties of CF-reinforced concrete. 

The fundamental motivation has been to strengthen the bond (1) among the carbon filaments within the roving, at least in the outer filament layers; and (2) between the carbon multifilament yarn (roving) and the cement-based matrix using a mineral, rather than a polymer coating. As a first step the sized carbon yarns should be treated with plasma, aiming at the formation of oxygen-bearing functional groups. Secondly, an aqueous suspension of mineral fines should be affixed to these partially hydrophilised yarns as a coating layer, which will later bring about the bonding to the cement-based matrix. Thirdly, these coated yarns should be incorporated into a concrete matrix and the mechanical behaviour should be assessed by pull-out tests. Morphological characterisation of the composite interphases, as well as the CF-matrix interface, will be used to relate them to the observations made in the mechanical tests.

## 2. Results and Discussion

### 2.1. Mechanical Performance: Pull-Out Tests

**Figure 1:**
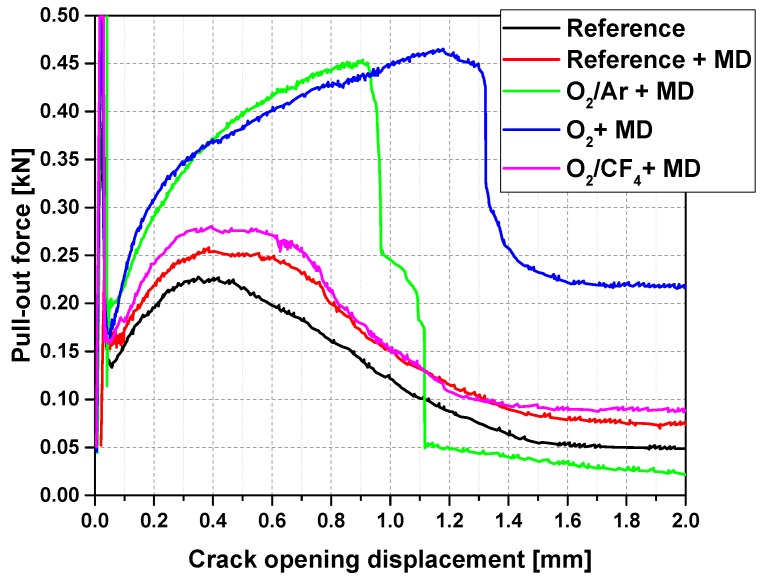
Representative pull-out force versus crack-opening-displacement curves for concrete specimens reinforced with a MD-coated and previously plasma-treated (for 100 s) CF roving; effect of various gas compositions can be seen and compared to reference samples.

Figure 1 displays the representative load-crack opening displacement curves for the reference specimens, as well as for the specimens reinforced with Mikrodur-coated (MD-coated) CF, which had undergone 100 s of plasma treatment with various gas compositions. 

**Figure 2:**
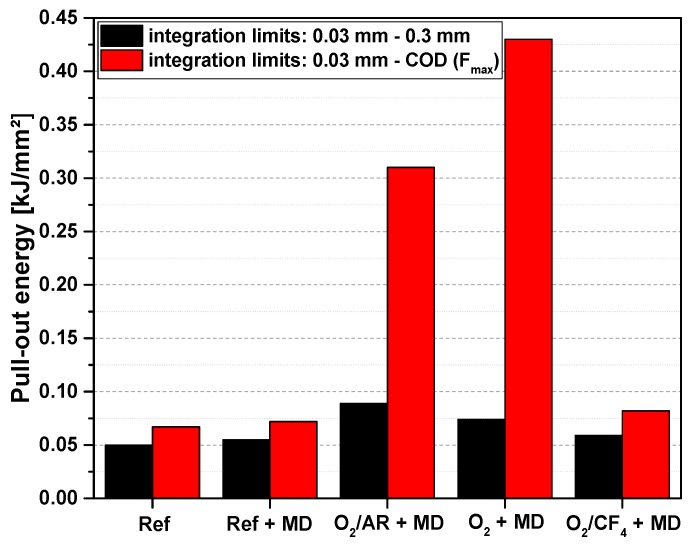
Typical pull-out energies calculated from the curves presented in Figure 1 for two ranges of crack opening displacement (COD): 0.03 mm to 0.3 mm (black columns) and 0.03 mm to COD at the maximum pull-out force (red columns).

At the beginning of the experiment, with deformations clearly below a mere 0.1 mm, each graph shows high transferred loads. This part of the curve represents the behaviour before and during crack formation in the concrete matrix at the notch. Hence, this part of the diagram was not taken into account when discussing any CF-concrete matrix interaction; see Figure 2 and Table 1. Beyond the low point of the sudden drop, the courses of the curves differed significantly, depending on the gas used for the plasma treatment. 

As expected, the reference reinforced by the CF with neither plasma treatment nor an MD-layer yielded the lowest pull-out force, of around 0.25 kN, at the lowest crack opening displacement of 0.43 mm; see Table 1. The subsequent gradual decrease in the pull-out force with further crack opening indicates a decreasing resistance to the CF pull-out from the concrete matrix; see Figure 1. This curve progression is characteristic of the insufficient load-bearing ability of the bond between the inherently non-polar CF reinforcement and the highly polar, water-based concrete matrix. The MD-coating of untreated CF resulted in a slight increase in the average maximum pull-out force, to 0.27 kN, at an average crack opening displacement of 0.51 mm (Table 1). Beyond the maximum value of the pull-out force-crack opening, the curves exhibit a narrow plateau before a gradual descent of the curve sets in; see Figure 1. Principally, such behaviour can be interpreted as fairly similar to the MD-free reference. The slightly higher maximum pull-force may be related to an expected increase in friction forces and interlocking, due to the fine MD-layer coating of the CF. The performance is still governed by CF pull-out from the concrete matrix, indicating rather poor CF/concrete and CF/MD bonding interactions.

Two out of the three types of plasma-treatment caused significant changes in the pull-out force versus the crack opening behaviour. The samples made of CF modified with plasma generated from pure oxygen or with a mixture of oxygen and argon, featured a dramatic increase in the transferred forces over a wide range of crack openings. For both of these types of plasma treatment, a pronounced ascent in the pull-out force of up to approximately 0.1 mm to 0.2 mm in the crack opening was observed, followed by a further moderate increase in the force with increasing crack opening displacement (Figure 1).

The CF treated with oxygen plasma exhibited an average maximum pull-out force of 0.50 kN, at a crack-opening displacement of 1.13 mm. After reaching the maximum pull-out force, a sharp drop in force was measured, followed by steady crack opening at a constant force. The samples made of CF modified with the plasma working with a mixture of oxygen and argon, showed an increase in the pull-out force up to 0.45 kN, at a 0.84 mm crack opening displacement; see Table 1. Subsequently, the force dropped steeply to the lowest value, indicating a full mechanical failure of the CF roving; cf. Figure 1. Apparently, both types of plasma treatment led to remarkably improved compatibility between the CF and the MD-coating and, finally, with the concrete matrix. Chemical and microstructural interpretation will be provided in Section 2.2. 

As a further plasma mixture, a combination of oxygen and tetrafluoromethane (O_2_/CF_4_) was applied to CF. Cement-based composite specimens containing this CF only exhibited a marginal increase in the maximum force, to 0.31 kN, in comparison to the reference samples (Table 1). The general shape of the corresponding force-displacement curve indicates no obvious success in activating the CF surfaces; cf. Figure 1. Potentially, the formed fragments in the plasma, e.g., CF_4_, could react with the carbon-based epoxy sizing layer by breaking the bonding and dissolving the carbon, which would result in an increased oxygen content at the surface. However, the effect was obviously negligible with respect to enhancing the CF-to-matrix bonding. Moreover, it should be noted that oxidation is suppressed by the effect of the fluorine atoms’ catching electrons and therefore prevents the formation of oxygen radicals and negative oxygen ions. 

The interpretation of the mechanical performance of the carbon concrete composite specimens was continued with respect to the pull-out energy calculated as the areas under the force-crack opening displacement curves; see Figure 2 and Table 1. For reasons of better comparability, each curve was integrated within two particular ranges. Firstly, the initial part of the curve was analysed. Therefore, integration occurred for crack-opening displacements between 0.03 mm and 0.3 mm, for any sample; see black columns in Figure 2. The lower limit of 0.03 mm represents the border between a simple matrix fracture and the onset of CF pull-out. Within the integration limits, i.e., 0.03 to 0.30 mm, both the references and the sample treated with the O_2_/CF_4_ plasma exhibited a pull-out energy between 0.05 kJ/mm^2^ and 0.06 kJ/mm^2^. The other two plasma-treated samples showed significantly higher values, of 0.075 kJ/mm^2^ (for O_2_ plasma) and 0.09 kJ/mm^2^ (for O_2_/Ar plasma). The increase in pull-out energy becomes much more pronounced when the upper limit is set to the crack opening displacement at the maximum load of each sample; see red columns in Figure 2. These integration limits were used for averaging and assessing the standard deviation as a statistical indicator of trustworthiness; see results in Table 1.

For the sample containing CF treated with pure oxygen plasma, a pull-out energy of 0.43 kJ/mm^2^ was calculated, which corresponds to an increase of more than four times, in comparison to the reference samples. The sample containing CF modified with the O_2_/Ar plasma also exhibited a high increase in pull-out energy (0.30 kJ/mm^2^, i.e., an increase of three times, compared to the reference). In contrast, the samples made with CF modified by the O_2_/CF_4_ plasma did not markedly differ from the reference samples. In addition to the qualitative curve shape, this result indicates a similar failure mechanism in these composites, which is dominated by friction between the CF and surrounding matrix, while the modification by oxygen plasma and oxygen/argon plasma resulted in an enhanced bonding between the components due to the introduction of polar groups (cf. Section 2.2).

**Figure 3:**
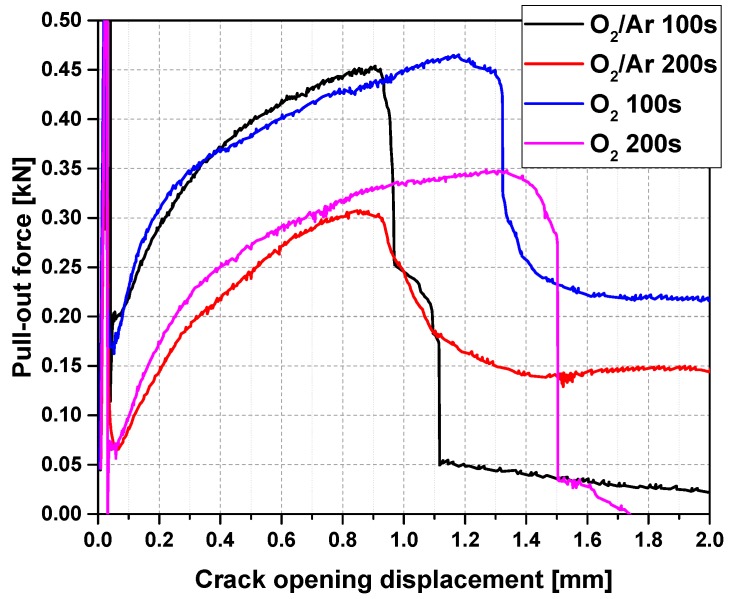
Typical pull-out force versus crack opening displacement for representative samples treated with plasma for 100 s and 200 s.

**Figure 4:**
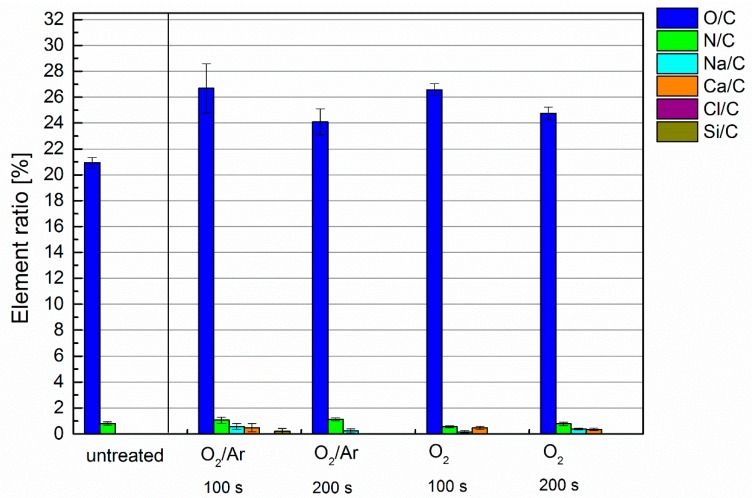
Element ratio of plasma-treated carbon fibres measured by X-ray photo electron spectroscopy; gas mixtures and treatment times are in accordance with Table 3.

For the two successful plasma treatments thus far, i.e., by using oxygen or the oxygen/argon mixtures, the treatment time was varied in the next step. Table 1 and Figure 3 disclose that in both systems, the maximum fibre pull-out force severely decreased in the case of the longer plasma treatment time of 200 s. By and large, the pull-out energies were nearly cut in half. However, the COD values at the maximum pull-out force did not change considerably, due to the prolonging of the plasma treatment. Despite the decreased maxima, the O_2_ plasma remained superior in comparison to the O_2_/Ar plasma, with respect to the positive effects of both on the mechanical performance of carbon concrete composites. The observed decrease in the maximum pull-out force can be explained by the enhanced deterioration of the epoxy resin by plasma-induced oxidation, which was advanced by longer treatment; an effect which also known for ceramic fibres [31]. As the results show and as could be expected, this influence is higher in the case of pure oxygen; see also Figure 4. An intensified oxidation can lead to a physical surface modification due to locally varying abrasion [32]. The physical condition of the surface governs the tensile properties of the CF roving, even prior to its coating with inorganic particles.

To elucidate in this respect, the tensile strength of the carbon fibre roving subject to respective plasma treatments was determined; see Table 2. The plasma-treated roving exhibits a tendency for higher tensile strengths, a finding previously reported by Dilsiz et al. [33]. However, such an effect could be explained by the procedure of sample preparation before plasma treatment. Owing to this, the roving experienced a stretching process, which results in an alignment of the individual CF filaments within the roving’s cross-section.

Consequently, a more uniform utilisation of all filaments was achieved in the case of mechanical loading and, hence, higher tensile strengths were achieved for CF. With an increasing duration of the plasma treatments, a decrease of 5% to 12% in the rovings’ tensile strengths occurred, which agrees with the results reported in the literature [34]. The longer the plasma treatment duration, the rougher the CF surface becomes [32], ending in the destruction of some CF filaments. Consequently, there seems to be an optimum treatment time which enables the wetting of CF by maintaining moderate sizing and avoiding damage to the CF filaments.

### 2.2. Physical, Chemical and Microstructural Interpretation of the Macro-Mechanical Performance of the CF-Reinforced Cement-Based Composites

From the results of mechanical testing, it can be concluded that the plasma process parameters play a crucial role in successful CF activation, enabling efficient load-bearing, a mineral-based coating of carbon roving, and ensuring an appropriate bond to the concrete matrix. The strikingly enhanced pull-out characteristics of the composites reinforced with oxygen, as well as the oxygen and argon plasmas, can be traced back to the presence of the oxygen-based functional groups on CF surfaces that had been introduced by these two plasmas. XPS results reveal a higher oxygen content at the surface of the carbon fibres after plasma treatment, both for oxygen, as well as for oxygen and argon as working gases, as compared to untreated CF; see Figure 4. The remarkably higher amounts of nitrogen on the fibre surfaces treated with the oxygen/argon plasma arise from the presence of nitrogen atoms and ions in this plasma. These reactive nitrogen species are induced by electrons, due to the electropositive effects of argon. On the other hand, oxygen has a high affinity for electrons, resulting in an electron-poor plasma that principally prevents the generation of nitrogen atoms or ions. Thus, the CF surface exhibits a lower nitrogen content when modified by an oxygen plasma. The presence of oxygen-based functional groups at the surface caused by treatment with oxygen-containing plasma results in a higher surface tension possessing a higher polar part [20,35]. This improves the binding interaction with the water-based slurry of MD and, eventually, with the reactive mineral particles.

**Figure 5:**
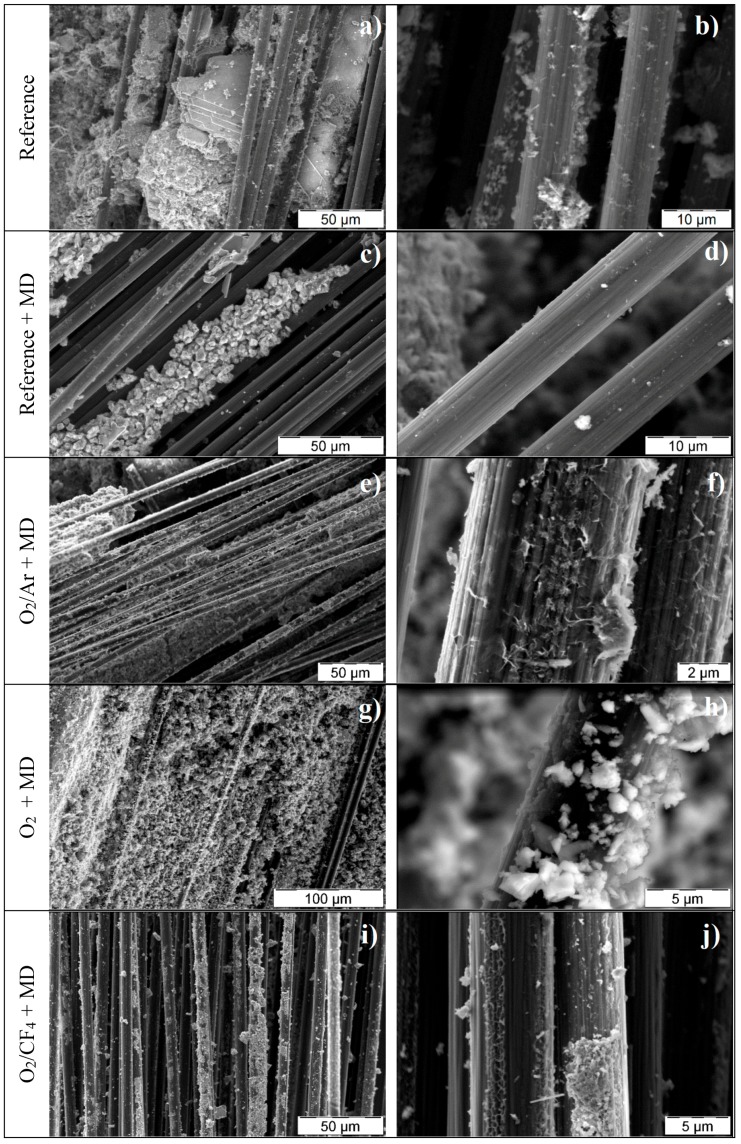
ESEM images of carbon fibre surfaces after the mechanical testing of the carbon-concrete-composite specimens (cf. Figure 11) showing different material residuals, depending on the type of plasma treatment (duration 100 s): (**a**,**b**) reference; (**c**,**d**) reference with MD; (**e**,**f**) O_2_/Ar and MD; (**g**,**h**) O_2_ and MD; (**i**,**j**) O_2_/CF_4_ and MD, each pairwise with lower and higher magnification.

**Figure 6:**
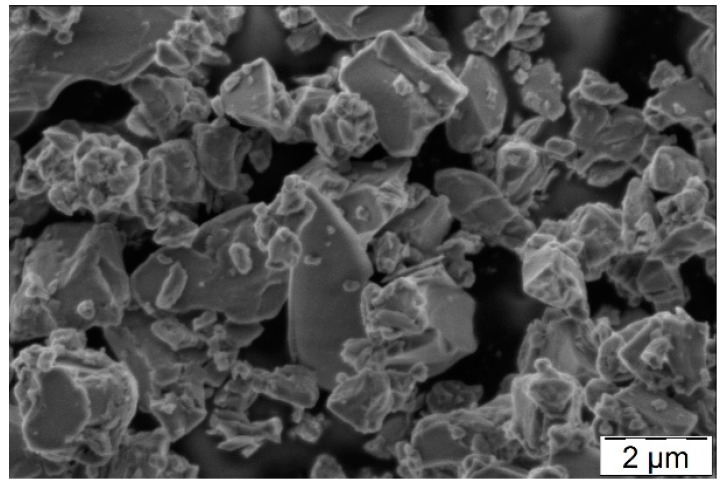
Morphology of MIKRODUR R-X showing sizes up to 4 µm in diameter and a typical appearance of slag-based mineral fines.

To substantiate the macro-mechanical findings from the viewpoint of the cement-based matrix, the composites were characterised morphologically and chemically-mineralogically. Figure 5 provides an overview of the morphology of the carbon filament surfaces after the tensile tests. When considering the reference samples, huge areas of uncoated surfaces lay bare (Figure 5a,b). Close to CF, large clumps of non-homogeneously distributed coating material were found, which obviously had no intense bond to any carbon surface. The reference samples with neither plasma treatment nor MD coating even displayed big portlandite crystals in the near vicinity of CF, due to the direct contact with the surrounding concrete matrix (Figure 5a). However, no hydration products exhibited bonding to any fibre surface. Some few, fine-structured hydration products can be seen locally, but only to a very minor extent. In the reference sample with the MD-layer (Figure 5c,d), clumped structures were present close to CF, showing no significant affinity towards them. Their appearance was similar to the initial MD morphology, having a typical structure for slag-based fines; see Figure 6. 

Moreover, at a higher magnification, the reference samples revealed only marginal areas of the carbon filaments’ coatings; see Figure 5. It can be concluded that without plasma treatment, no affinity between CF filaments and MD matrix or fine-grained concrete matrix occurred. The hydrophobicity of unmodified CFs did not allow any growth of mineral structures on the surface. This finding explains the poor mechanical performance, as obtained from the pull-out tests. Some slight increase in pull-out forces may result from more pronounced friction and interlocking due to the MD coating. However, the missing electrostatic interaction between the constituents of the water-based mineral matrix and the inherently hydrophobic CF does not enable any considerable enhancement of load transfer between CF and the concrete matrix.

In contrast, the morphological appearance of any embedded plasma-treated CF was found to be fundamentally different to both untreated references. They possess prominent areas of mineral accretions of tightly bonded, finely structured hydration products (especially in cases when O_2_/Ar plasma and O_2_/CF_4_ plasma treatment), as well as residual MD particles (especially in case of O_2_ sample) on each single filament’s surface (Figure 5e–j).

Among these plasma-treated specimens, the CF samples treated with O_2_/CF_4_ plasma had the most non-uniform CF coverage by such inorganic material (Figure 5i,j). The partially dense coating of CF filaments alternated with vast bare, uncoated CF areas. Such morphology explains the modest mechanical performance of this composite under tensile loading, with force-crack opening displacement curves similar to the curves obtained for the reference specimens without plasma treatment. There are too few build-ups bonded to CF surfaces, so that each filament can be pulled out smoothly from the surrounding concrete matrix. In contrast, the composite specimens reinforced with roving treated by O_2_ plasma and O_2_/Ar plasma featured numerous, large regions of mineral structures growing on the filament surface. These formations give rise to a superior mechanical performance during crack opening, as the consequence of both a high coating density and a very high number of bonding points of the minerals to the yarn. Thus, the surface of each single filament has an intense linkage to the matrix. The bonded mineral layer enables load transfer from the concrete matrix onto each single CF, resulting in increased load bearing capacity. Hence, these composites featured a fundamentally different pull-out behaviour in comparison to the reference samples. Interestingly, the ESEM images suggest that the prevailing minerals attached to the CF surfaces differ in the cases of the oxygen and the oxygen/argon plasmas. On the oxygen-treated filaments, MD grains prevail, whereas typical hydration products from cement and pozzolana are present, only to a lesser extent. Contrarily, more of these hydration products seem to be present on CF surfaces treated with O_2_/Ar plasma in comparison to the extent of unreacted or partially reacted MD grains. The hydration products are fine-structured interpenetrating needles, and their morphology strongly suggests that, chemically, they are intensely bonded to the CF surface. It appears that they originate from the interface, demonstrating heterogeneous nucleation, rather than having been precipitated out of solution. Furthermore, the remaining MD grains give the impression of an intense adhesion to the surface of each filament. Unlike in the case of the reference (no plasma treatment), no portlandite was found in the interphase layers of the composite specimens containing CF treated with oxygen and oxygen/argon plasmas, the reason for which being the extensive pozzolanic reaction with the locally available slag-based MD grains. 

**Figure 7:**
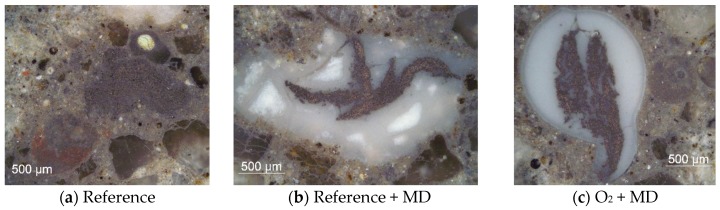
Optical micrographs of the cross-sections showing a carbon fibre roving (**a**) without mineral coating; (**b**) with mineral coating; and (**c**) with oxygen plasma treatment and mineral coating, all embedded in the concrete matrix.

**Figure 8:**
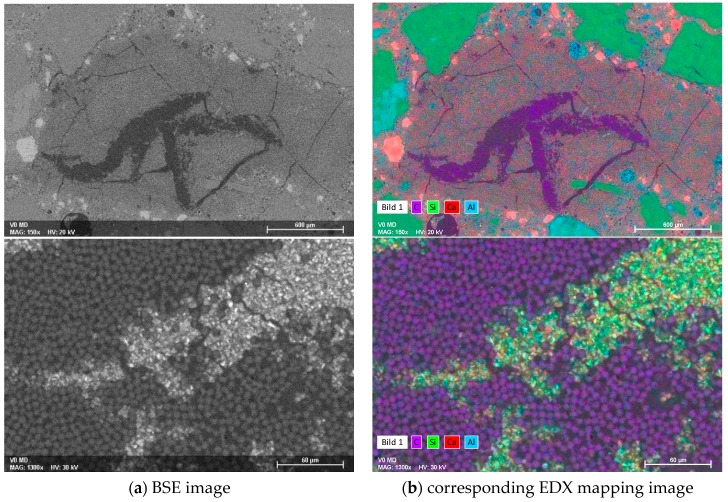
(**a**) ESEM BSE images of different magnification; and (**b**) corresponding EDX mapping of a representative cross-section of a plasma untreated CF roving sample with an MD layer embedded into the concrete matrix.

**Figure 9:**
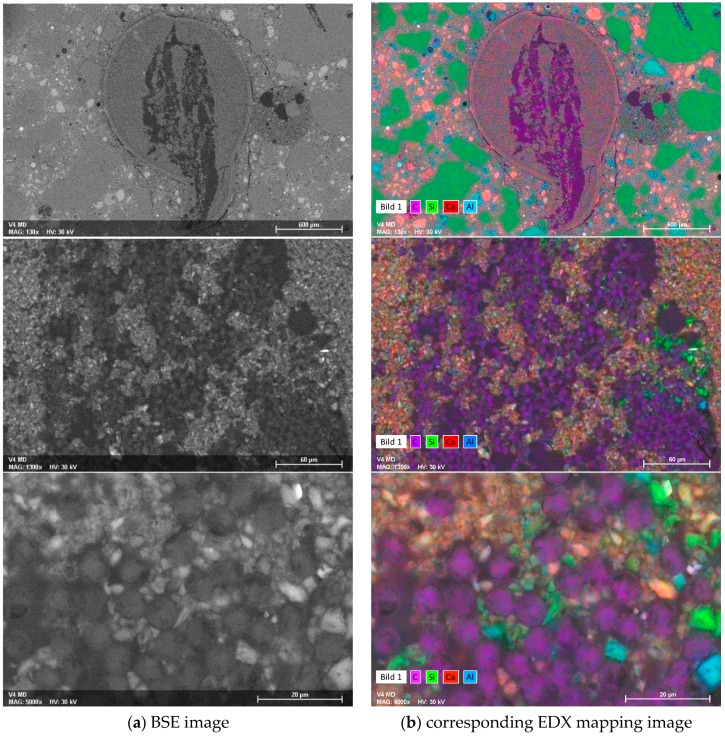
(**a**) ESEM BSE images of different magnification; and (**b**) corresponding EDX mapping of a representative cross-section of oxygen-treated plasma CF sample with an MD layer embedded into the concrete matrix.

The distinct morphological differences are even more evident in the readily prepared cross-sections of the samples; see Figure 7, Figure 8 and Figure 9. Figure 7 presents selected optical micrographs. None of the CF roving featured a circular or elliptical shape. Instead, irregular shaped roving cross-sections were distributed non-uniformly within the surrounding concrete matrix. As a result, all of the samples possessed various conditions of CF-matrix interfaces. In both reference samples (no plasma treatment), the multifilament strands were embedded rather compactly in the concrete matrix or in the MD, as an adjacent layer. The cement-based matrix or MD had only marginally inserted itself between the CF filaments. In contrast, significant amounts of MD were present throughout the entire cross-section of the multifilament yarns treated with the O_2_ plasma. Additionally, a dark-stained ring around the coating layer is to be observed, induced by a densification effect.

ESEM images of the cross-sections demonstrate the described features even more clearly, and by additional EDX elemental mapping, mineralogical details were elucidated; see Figure 8 and Figure 9. Figure 8 shows the back-scattered electron (BSE) images and the corresponding EDX mapping of the reference samples with an MD coating. At a lower magnification (upper images), the heterogeneous structure of CF (coloured in purple, representing carbon), the surrounding MD layer, and the embedment of that into the concrete matrix, are clearly visible. Moreover, shrinkage cracks can be observed within the MD layer, which appear in the upper EDX image in purple, due to the introduction of epoxy resin into the preparation process. The higher magnification shows a densely packed CF roving, in which only few, rather large cross-sectional areas with interpenetrated MD in the middle of the filament bundle are present. The majority of the single filaments’ “neighbours” are further carbon filaments, but neither MD nor the cement-based matrix are in the close vicinity. Hence, the filaments are adjacent to each other in a large proportion, without adhesion-promoting linkage by the mineral matrix.

This gives rise to sliding pull-out without any significant load transfer over the cross-section of the roving. The poor bond properties result in modest mechanical characteristics of carbon-concrete-composites containing CF without plasma treatment. In contrast, the samples treated with O_2_ plasma exhibited much more pronounced infiltrated MD areas within the CF roving; see Figure 9. 

The lower magnification already visualises a uniform distribution of carbon filaments (coloured in purple) within the surrounding mineral matrix. Higher magnifications strikingly disclose MD and matrix infiltration all over the cross-section of the multifilament yarn. The fibre/mineral interface was situated not only on the outer filaments of the roving, but was also spread inwardly, to the majority of the inner filaments. This enables a significantly better load transfer from the concrete matrix towards the coated carbon filaments, thus explaining the superior mechanical performance of such composites in the pull-out tests. 

At this point, it is useful to recall the visual impressions during the experimental execution of the dip-coating. The MD slurry and the O_2_-plasma-treated CF must have been very affine, because the slurry was strongly drawn into non-submerged regions, which was not the case for the plasma-untreated CF samples during dipping. These findings and observations further underpin the notion that the oxygen-containing plasma induced the collection of oxygen-based polar groups on the CF surface, thus enabling distinctively better wetting [21,24]. Furthermore, the bonding force to mineral particles suspended in the liquid phase greatly increased.

## 3. Materials and Methods 

### 3.1. Sample Preparation

**Figure 10:**
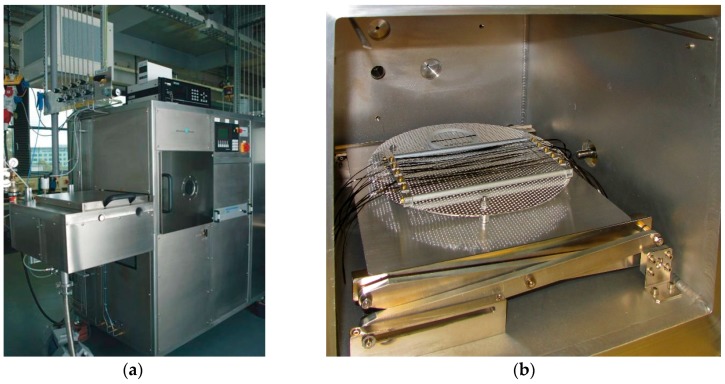
(**a**) The plasma chamber, as installed and operated at INP Greifswald; (**b**) Interior of the plasma chamber with the CF rovings mounted on a frame.

A commercially available sample of CF roving with an epoxy-based sizing (Toho Tenax 6K, yarn fineness 400 tex, Toho Tenax Europe GmbH, Wuppertal, Germany) was selected for this fundamental study and used as obtained. A cubic plasma chamber (400 mm × 400 mm × 400 mm) was used for all processes (Figure 10a). Pieces of the roving were mounted on a frame (Figure 10b) and placed in the plasma chamber, so that overall treatment of every outer CF surface was ensured. Treatment of the fibres was performed under low pressure conditions (100 Pa), using pulsed microwave discharges (pulse: 10 ms on, 90 ms off). The discharge power was set at 1200 W. A vacuum was applied, before inserting the gas source and adjusting the pressure. Three different reactive gases or gas mixtures were used for the plasma-based modification of the carbon fibre surfaces: oxygen, oxygen and argon, and oxygen and tetrafluoromethane CF_4_ (Table 3). Additionally, the exposure times were varied; they were set at 100 s and 200 s, respectively.

The plasma-treated carbon fibres were investigated, with respect to their surface chemistry, by X-ray photo electron spectroscopy (XPS; Axis Ultra, Kratos, Manchester, UK). The maximum energy resolution was 0.45 eV (Ag 3d) and the local resolution was in the range of 7 μm. The information depth averaged 10 nm, with a detection limit of 0.1% to 1%. The hydrophilic properties were determined by contact angle measurements (Surftens, OEG GmbH, Frankfurt (Oder), Germany). Moreover, the tensile strengths of the CF rovings were measured according to DIN 3341. 

Immediately following the plasma treatment, the mineral coatings were applied. The commercially available powder blend, Dyckerhoff MIKRODUR R-X, abbreviated as MD, based on granulated blast furnace slag [36] and produced by Dyckerhoff Beton GmbH & Co. KG, Wiesbaden, Germany, was used as the mineral coating material. It was manually mixed with tap water in a mass proportion of one-to-one, to obtain a homogenous suspension with a high workability. The freshly plasma-treated CFs were dip-coated by hand. They were immersed to the point of saturation by capillary suction forces for roughly 30 s, to ensure the best possible overall coating. No additional mechanical impregnation was performed. Subsequently, the coated yarns were dried by draping them over a clothesline in the standard atmosphere of the concrete laboratory (20 °C, 65% relative humidity) for seven days, before their incorporation into the concrete matrix.

The composition of the concrete matrix is summarised in Table 4. The cement was a normal Portland cement CEM I 32.5 R (Schwenk, Bernburg, Germany), according to EN 197-1. As pozzolanic additives, a hard-coal fly ash (Steament H-4, STEAG Power Minerals GmbH, Essen, Germany) and silica fume in the form of an aqueous slurry EMSAC 500 SE from BASF, Ludwigshafen, Germany, were used. A local quartz sand was obtained from Kieswerk Ottendorf-Okrilla, Ottendorf-Okrilla, Germany. To ease the workability, the β-naphthalene sulfonate (BNS) high-range water-reducing admixture (HRWRA) MasterRheobuild 30 from BASF, Germany, was added as received.

The fine-grained concrete was prepared in a 5 L planetary mixer (HSM20, Hobart, Leipzig, Germany). Each batch had an overall volume of 2 L. Any dry components such as cement, fly ash, and sand were pre-blended for 1 min in the first gear. The liquid components, i.e., silica slurry, HRWRA, and water, were added and mixed for 1 min, also in first gear. Subsequently, stirring was stopped, and residual unmixed materials were removed from the bucket walls and added to the mixture. Homogenisation was continued for an additional 2 min in the second gear.

**Figure 11:**
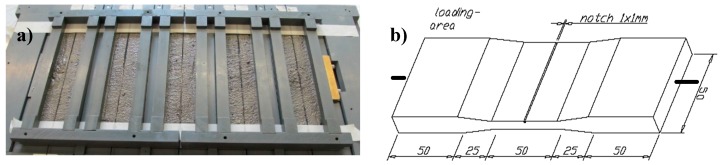
(**a**) half-filled moulds with coated fibre in place; and (**b**) sample dimensions (in mm).

For each plasma treatment (cf. Table 3), five replicate carbon concrete composite specimens were prepared and characterised. One CF-reinforced concrete specimen series containing MD-coated carbon roving, but without prior plasma treatment, served as a reference (five replicates). Another reference composite, from which ten individual specimens were taken, was made of the virgin CF, with neither plasma treatment nor MD-coating. CF-reinforced composites were prepared in accordance with Figure 11a, in moulds having the specific dimensions displayed in Figure 11b. The formwork introduced a notch of 1 mm × 1 mm to initiate locally defined matrix cracking under tensile loading. After filling the first half of the mould with the matrix, the roving was placed in the centre of the specimen, along its longitudinal axis. Subsequently it was covered with a second layer of fresh, fine-grained concrete. Further formwork imprinting the specific geometry into the upper half of the specimen was tightly clamped on top. Neither vibrating nor densifying and, hence, no further energy input, was applied. The samples were kept for one day in the tight mould and were subsequently immersed in water for six days, according to DIN EN 12 390-2, followed by storage at a standard laboratory climate (20 °C, 65% relative humidity) until mechanical testing. 

### 3.2. Mechanical Tests, Morphological and Analytical Characterisation

**Figure 12:**
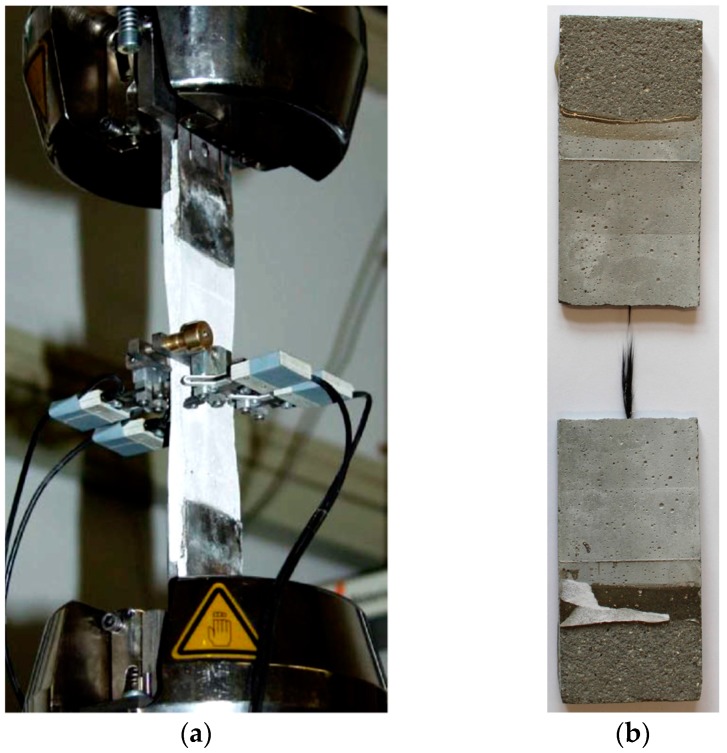
(**a**) Setup for uniaxial quasi-static double-sided pull-out tests, according to [37]; (**b**) sample after testing.

Mechanical characterisation was performed as uniaxial quasi-static double-sided pull-out testing, according to the setup and procedure described by Butler et al. [37]. Notched specimens were tested at an age of 28 days. Five replicate specimens were produced and tested per material composition. A universal servo-hydraulic machine (Instron 8501, Instron, Darmstadt, Germany) equipped with a UK2518 load cell (max. 10 kN) was used under a crosshead displacement-control regime (Figure 12), with a displacement rate of 1 mm/min. The specimen (Figure 12) was clamped at both ends. Applied load versus crack-opening displacement, measured using an extensometer; cf. Figure 12a, was recorded. To ease visual perception, only typical, representative single-test curves are displayed for each parameter combination under investigation. Additionally, the average values of the maximum recorded load and of the crack opening displacement at the maximum load for the replicate specimens were calculated, along with the respective standard deviations. Furthermore, the pull-out energy was determined as the area under the individual load-displacement curves. Among replicate specimens, the pull-out energies were averaged and the standard deviations were calculated. Details of integration borders are provided and explained in Section 2.1.

Morphological characterisation was comprised of the assessment of MD-coated CF and CF-reinforced concrete specimens. In the latter case, two alignments per specimen were examined: (1) the specimens were split in the direction of the fibre; (2) cross-sections were prepared by infiltration with an epoxy resin to later characterise the intergrowth among CF, the MD coating, the concrete matrix, and the hydration products. The tools of instrumental analysis were an environmental scanning electron microscope (ESEM; Quanta 250 FEG, FEI, Eindhoven, The Netherlands), with which each specimen was inspected without further preparatory steps, an energy-dispersing X-ray analyser (EDX; Quantax 400, Bruker, Billerica, MA, USA) for element mapping, and an optical microscope (AXIOTECH, Zeiss, Jena, Germany) equipped with a live camera (AxioCam ICc 3, Zeiss, Jena, Germany).

## 4. Summary and Conclusions

In this investigation, the plasma treatment of carbon multifilament roving was successfully applied for enhanced interaction with a mineral-based coating and a subsequently improved load transfer to the concrete matrix. 

By using three various process plasmas, different interfacial bonding characteristics towards the mineral coating were observed, by accomplishing mechanical pull-out tests on carbon-concrete-composite specimens. The CF treated with pure oxygen plasma showed the most improvement in the mechanical performance in this test, followed by the CF treated with oxygen/argon plasma. An increase in the pull-out energy measured up to the load maximum exhibited a rise of four times and three times the value of the references samples, respectively. In contrast, only a slight increase was detected for the CF treated with O_2_/CF_4_ plasma.

The striking differences in the mechanical performance observed for treated and untreated carbon fibres were explained by means of morphological characterisation using ESEM/EDX and optical microscopy. A superior mechanical performance was observed for carbon roving with a uniform coating, which impregnated the interior of the roving and was well bonded to the carbon filaments. Such a coating was unambiguously mediated by the functional groups on the CF surface introduced by plasma treatment, which enabled chemical bonding between the carbon filaments and the mineral coating. In contrast, mostly bare and uncoated CFs were found in the reference samples, which were not plasma-treated. They showed no interaction with the mineral coating or with the hydration products of the fine-grained concrete matrix. 

The samples treated with oxygen plasma exhibited an intense impregnation of the mineral binder grains of the coating suspension between the carbon filaments, induced by the strong affinity of the functionalised CF surface and the polar, water-based slurry. This resulted in a pronounced increase in the CF-mineral coating interface and in enhanced bonding between the components, i.e., CF-to-coating and coated roving-to-concrete matrix, thus enabling a persistent mechanical load transfer between carbon yarn. This explains the superior mechanical performance of the composite specimens containing plasma-treated carbon roving in comparison to the reference specimens containing untreated carbon roving. 

Along with the plasma source, the duration of treatment was found to be crucial. With a longer treatment time, the mechanical performance in the pull-out experiments severely decreased. This can be traced back to advanced oxidation and, therewith, partial damage to the carbon fibre. 

It can be concluded that plasma-driven functionalisation of sized-only carbon roving is a very promising method for promoting the fibre-matrix bond in carbon concrete composites, without introducing a petrochemical impregnation of the carbon strands. The ongoing work of the authors deals with the systematic optimisation of plasma treatment, developing new compositions for mineral coatings, its application technology, and testing the performance of such carbon concrete composites under elevated temperatures.

## Figures and Tables

**Table 1:** Average values and standard deviations of maximum CF pull-out forces, the corresponding crack opening displacements, and the pull-out energy among replicate specimens (numbers in brackets indicate the standard deviation).

Specimen Series	Maximum CF Pull-Out Force (kN)	Crack Opening Displacement at Maximum CF Pull-Out Force (mm)	Pull-Out Energy (kJ/mm^2^)
reference w/o MD	0.25 (0.03)	0.43 (0.14)	0.09 (0.04)
reference with MD	0.27 (0.03)	0.51 (0.08)	0.10 (0.03)
O_2_/Ar (100 s)	0.45 (0.07)	0.84 (0.10)	0.30 (0.07)
O_2_/Ar (200 s)	0.30 (0.06)	0.79 (0.16)	0.18 (0.07)
O_2_ (100 s)	0.50 (0.05)	1.13 (0.19)	0.43 (0.08)
O_2_ (200 s)	0.35 (0.06)	0.95 (0.19)	0.24 (0.07)
O_2_/CF_4_	0.31 (0.07)	0.61 (0.24)	0.15 (0.09)

**Table 2:** Tensile strength of the carbon fibre roving subjected to various plasma treatments.

CF Roving	Number of Specimens Tested	Mean Value (MPa)	Standard Deviation (MPa)	Variance (%)
reference	10	1472.6	159.5	10.8
O_2_/Ar (100 s)	5	1734.2	80.9	4.7
O_2_/Ar (200 s)	5	1531.8	107.4	7.0
O_2_ (100 s)	5	1554.7	217.9	14.0
O_2_ (200 s)	5	1479.1	98.9	6.7

**Table 3:** Sample denomination and variation of plasma treatment parameters.

Sample Denomination	Gas	Gas Flow (mL/min)	Plasma Exposure Time (s)
reference	-	-	-
O_2_/Ar (100 s)	O_2_/Ar	10/5	100
O_2_/Ar (200 s)	O_2_/Ar	10/5	200
O_2_ (100 s)	O_2_	10	100
O_2_ (200 s)	O_2_	10	200
O_2_/CF_4_	O_2_/CF_4_	100/5	100

**Table 4:** Fine-grained concrete composition and its characteristics in both the fresh and hardened states.

Material	Unit	Value
cement (CEM I 32.5 R)	kg/m^3^	548.8
fly ash (class F)	kg/m^3^	245.6
silica slurry (aqueous suspension, solids content 1:1 wt. %)	kg/m^3^	54.6
binder content (cement + fly ash + silica fume)	kg/m^3^	821.7
sand (0–1 mm)	kg/m^3^	1091.5
superplasticizer (solution as obtained)	wt. % of binder	2.3–2.4
tap water	kg/m^3^	245.6
*w*/*c*	-	0.45
*w*/(cement + fly ash + silica fume)	-	0.30
slump flow (test according to DIN 18555-2)	mm	180–200
average compressive strength at the age of 28 days (test according to DIN EN 196-1)	MPa	63.5
average flexural strength at the age of 28 days (test according to DIN EN 196-1)	MPa	8.6
shrinkage after 28 days (test according to DIN 52450)	mm/m	0.83
shrinkage after 360 days (test according to DIN 52450)	mm/m	1.27
